# Fractional fetal thigh volume in the prediction of normal and abnormal fetal growth during the third trimester of pregnancy

**DOI:** 10.1016/j.ajog.2017.06.018

**Published:** 2017-10

**Authors:** Louise E. Simcox, Jenny E. Myers, Tim J. Cole, Edward D. Johnstone

**Affiliations:** a Maternal and Fetal Health Research Center, Institute of Human Development, University of Manchester, Manchester, United Kingdom; b St Mary’s Hospital, Central Manchester University Hospitals National Health Service Foundation Trust, Manchester Academic Health Science Center, Manchester, United Kingdom; c Great Ormond Street Institute of Child Health, University College London, London, United Kingdom

**Keywords:** estimated fetal weight, fetal growth restriction, fractional thigh volume, small for gestational age, 3-dimensional ultrasound

## Abstract

**Background:**

Currently, 2-dimensional ultrasound estimation of fetal size rather than fetal growth is used to define fetal growth restriction, but single estimates in late pregnancy lack sensitivity and may identify small for gestational age rather than growth restriction. Single or longitudinal measures of 3-dimensional fractional thigh volume may address this problem.

**Objective:**

We sought to derive normal values for 3-dimensional fractional thigh volume in the third trimester, determine if fractional thigh volume is superior to 2-dimensional ultrasound biometry alone for detecting fetal growth restriction, and determine whether individualized growth assessment parameters have the potential to identify fetal growth restriction remote from term delivery.

**Study Design:**

This was a longitudinal prospective cohort study of 115 unselected pregnancies in a tertiary referral unit (St Mary’s Hospital, Manchester, United Kingdom). Standard 2-dimensional ultrasound biometry measurements were obtained, along with fractional thigh volume measurements (based on 50% of the femoral diaphysis length). Measurements were used to calculate estimated fetal weight (Hadlock). Individualized growth assessment parameters and percentage deviations in longitudinally measured biometrics were determined using a Web-based system (iGAP; http://iGAP.research.bcm.edu). Small for gestational age was defined <10th and fetal growth restriction <3rd customized birthweight centile. Logistic regression was used to compare estimated fetal weight (Hadlock), estimated fetal weight (biparietal diameter–abdominal circumference–fractional thigh volume), fractional thigh volume, and abdominal circumference for the prediction of small for gestational age or fetal growth restriction at birth. Screening performance was assessed using area under the receiver operating characteristic curve.

**Results:**

There was a better correlation between fractional thigh volume and estimated fetal weight ((biparietal diameter–abdominal circumference–fractional thigh volume) obtained at 34-36 weeks with birthweight than between 2-dimensional biometry measures such as abdominal circumference and estimated fetal weight (Hadlock). There was also a modest improvement in the detection of both small for gestational age and fetal growth restriction using fractional thigh volume–derived measures compared to standard 2-dimensional measurements (area under receiver operating characteristic curve, 0.86; 95% confidence interval, 0.79–0.94, and area under receiver operating characteristic curve, 0.92; 95% confidence interval, 0.85–0.99, respectively).

**Conclusion:**

Fractional thigh volume measurements offer some improvement over 2-dimensional biometry for the detection of late-onset fetal growth restriction at 34-36 weeks.

## Introduction

The detection of fetal growth restriction (FGR) antenatally remains a challenge,^1^ as undetected abnormalities in fetal growth remain one of the strongest risk factors for stillbirth and term perinatal death.^2^, ^3^, ^4^, ^5^, ^6^ This problem is particularly important in late-onset FGR, which is usually defined by ultrasound estimation of fetal size; however, single estimates of fetal size have a low sensitivity for the detection of FGR.^7^ It is also difficult to distinguish between a fetus that is constitutionally small for gestational age (SGA) and one that has pathological FGR. This is important in clinical practice as it is recognized that growth-restricted fetuses are most at risk for adverse perinatal outcomes such as admission to neonatal intensive care unit, low 5-minute Apgar scores, neurological injury, and even stillbirth or early neonatal death.^8^, ^9^ Many different methods have been described to differentiate between healthy and pathologically small fetuses including the use of customized growth charts,^10^ analysis of placental and fetal Doppler blood flow,^11^ analysis of fetal growth velocity,^12^ and the use of placentally derived biomarkers.^13^ However, these methods have been evaluated mainly in the context of early-onset FGR <34 weeks.^14^ Doppler ultrasound of umbilical artery flow, which is the mainstay of FGR diagnosis and management in early-onset FGR, is of limited use in identifying term FGR, as this will often be normal.^14^ Screening using the cerebroplacental ratio and uterine artery pulsatility index at 35-37 weeks has demonstrated potential, but this requires further validation.^15^, ^16^, ^17^, ^18^, ^19^

Despite the limitations of fetal size–based assessment, investigators have continued to examine the use of estimated fetal weight (EFW) calculated using 2-dimensional (2D) ultrasound measurements such as that described by Hadlock et al.^20^ This screening is based on estimations of fetal size at a single point rather than growth velocity, so that fetuses >10th centile, but not achieving their growth potential, will not be identified as at risk of FGR. Unsurprisingly, detection of SGA is better the nearer the ultrasound scan is performed to delivery (70-75% at 35-37 weeks and 50-60% at 30-34 weeks for the same 10% false-positive rate).^21^ These single estimates of fetal size are also hampered by the lack of adjustment for differences in individual growth potential and therefore a method designed to adjust for individual growth was developed: individualized growth assessment (IGA).^12^ In IGA, second-trimester growth velocity data are used to determine Rossavik growth models that predict individual third-trimester growth trajectories. Three-dimensional (3D) sonography may allow more accurate assessment of fetal weight and improved differentiation between normal and pathological growth because it includes soft-tissue volume.^22^ Fetal thigh volume and the derived fractional thigh volume (TVol) were reported to be the most accurate and reproducible method to estimate birthweight.^23^, ^24^, ^25^, ^26^ Second-trimester TVol measurements can also be used to generate Rossavik models for predicting TVol and EFW growth trajectories during the third trimester.^27^, ^28^

The aim of this study was to create reference centiles for TVol measurements and to examine whether 3D measurements of fetal soft tissues can detect pathological deviations in fetal growth more accurately than conventional 2D measurements.

## Materials and Methods

### Study design

This was a longitudinal prospective study of fetal biometry using 2D and 3D ultrasound in 1 tertiary referral unit (St Mary’s Hospital, Manchester, United Kingdom). From November 2013 through July 2015, women with healthy uncomplicated singleton pregnancies were invited to participate. Exclusion criteria were: fetuses subsequently shown to have a major congenital abnormality, multiple pregnancies, and maternal medical conditions known to affect fetal growth such as maternal diabetes, renal disease, and chronic hypertension. Ultrasound scans were performed for research purposes and were not part of routine antenatal care, and did not include clinically indicated examinations. Scan results were available to view in the clinical records.

### Participant recruitment

**Figure 1:**
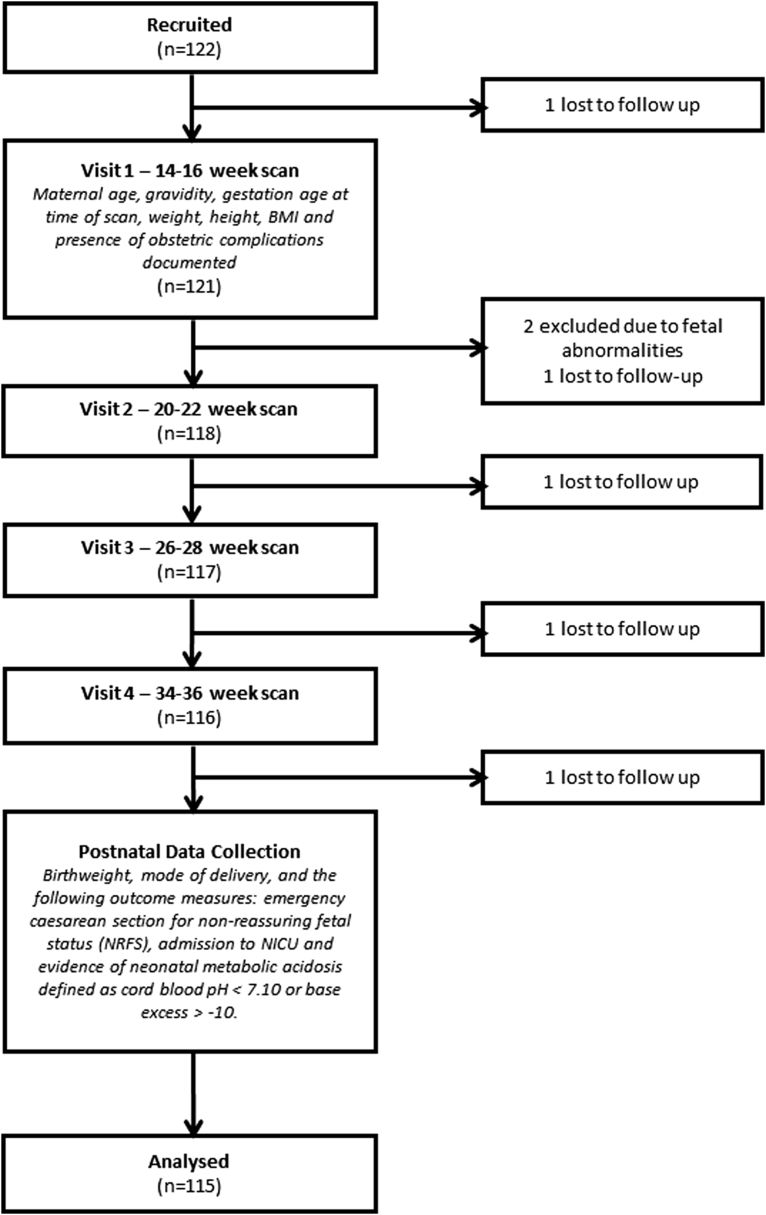
Study design and participant recruitment and follow-up flow diagram Participant flow diagram. *BMI*, body mass index; *NICU*, neonatal intensive care unit. *Simcox et al. Fractional thigh volume use in third-trimester fetal assessment. Am J Obstet Gynecol 2017*.

The study was approved by the Greater Manchester East National Research Ethics Committee in 2010 (Ref 10/H1013/9) and all participants were enrolled under signed informed consent. Women meeting study criteria were approached, and given verbal and written information when they attended for their booking 12-week scan appointments. Pregnancies were dated using fetal crown-rump length measurements <14 weeks’ gestation.^29^ In all, 122 women were recruited to the study; all pregnancies were prospectively monitored and clinical information was collected using a questionnaire during each scan visit and after delivery (Figure 1).

### Ultrasonographic data collection

All 2D and 3D ultrasonography was performed by 2 Royal College of Obstetricians and Gynecologists–accredited sonographers (L.E.S. and E.D.J. [Royal College of Radiologists accredited]) with ≥4 years of experience with obstetric ultrasound,^30^ using a Voluson E6 (GE Healthcare, Chicago, IL) with 3D 4- to 8-Hz curvilinear probe. The study protocol included a scan for 2D fetal biometry (biparietal diameter [BPD], head circumference [HC], abdominal circumference [AC], femur length [FL]), and 3D TVol at 14-16, 20-22, 26-28, and 34-36 weeks’ gestation. EFW (Hadlock) was calculated as: log_10_ EFW = 1.335 – (0.0034 AC × FL) + 0.0316 BPD + 0.0457 AC + 0.1623 FL.^20^ EFW centile was calculated using Hadlock et al.^20^, ^31^ Participants were not excluded from the study if growth abnormalities were detected at the later scans, but underwent closer monitoring of fetal growth. Clinicians were not blinded to clinical information/women’s history at the time of examination, and both participants and clinicians were not blinded to the results of the 2D measurements as these appeared on the screen and were reported in the woman’s notes at the time of the scan. Automated volume measurements were obtained using the Voluson 4D view tool (GE Healthcare). TVol was calculated as described by Lee et al.^32^ The 3D images were archived for later analysis in random order with clinicians blinded to patient identity. Each research scan took 20 minutes with postimage analysis for TVol measurements taking 5 minutes for each volume. Reproducibility of TVol measurements has previously been examined in 25 women in the second trimester and 33 women in the third trimester, and across both trimesters in 40 women in separate studies.^30^, ^32^ The intraclass correlations were 0.992 for intraexaminer variability and 0.943 for interexaminer variability.^30^

### Generation of TVol centiles

Reference centiles for TVol from 14-37 weeks’ gestation were produced using the LMS method based on 357 scans from fetuses with normal birthweight outcomes (normal growth potential and birthweight >10th centile).^33^ This method summarizes the changing distribution of a measurement with age by 3 curves representing the median (M), coefficient of variation (S), and skewness (L), the latter expressed as a Box-Cox power. Using penalized likelihood, the 3 curves can be fitted as cubic splines by nonlinear regression, and the extent of smoothing required can be expressed in terms of smoothing parameters or equivalent degrees of freedom.^33^ A key assumption of the method is that skewed data can be rendered normal with suitable power transformations. The analysis was performed using the computer software LMSchartmaker.^34^ Fitted L, M, and S curves were obtained by setting the equivalent degrees of freedom to 1, 8, and 4, respectively, and measurements were converted to centiles using LMSgrowth.^35^

### Biometry analysis

All data were assessed for normality of distribution by visual inspection of histograms and the Shapiro-Wilk test. Positively skewed data were transformed to base 10 logarithms. Linear regression analysis was used to assess the relationship with birthweight of 2D and 3D ultrasound measures at 26-28 and 34-36 weeks’ gestation, adjusting for gestational age at the time of the scan and at delivery. Hindmarsh et al^36^ reported that 2D biometry at 30 weeks’ gestation explained 40% of the variance in birthweight. To assess the relationship with birthweight using 2D and 3D biometry obtained at 34-36 weeks, a sample size of 112 patients was calculated as sufficient to identify a 20% increase in the variance explained (from 40% for 2D to 60% for 3D), assuming a type 1 error of 5% and power of 80% using a Fisher 1 correlation z test.

SGA was defined as final customized birthweight (individual birthweight ratio [IBR]) <10th centile and FGR final customized birthweight (IBR) <3rd centile using Gestation-Related Optimal Weight (GROW) software (Version 6.7.6.1_15[UK]).^37^, ^38^ Logistic regression was used to compare the utility of 2D and 3D ultrasound measures (EFW [Hadlock], EFW [BPD–AC–TVol], AC, TVol) to predict SGA and FGR. The EFW prediction model that was used for BPD, AC, and TVol was based on a previous study by Lee et al^26^, ^39^ and a subsequent validation study. Screening performance was compared using the area under the receiver operating characteristic curve (AUC) (method of DeLong et al^40^). This analysis was performed on 115 women with complete data. Using data from Roma et al,^41^ we calculated that 84 participants would be needed to detect a change in the detection of FGR from 60% for 2D to 72.5% for 3D (power 80%, type I error 5%).^42^ Statistical analysis was performed using SPSS, Version 22 (IBM Corp, Armonk, NY); Stata 13.1 (StataCorp, College Station, TX); and GraphPad Prism 6 for Windows (GraphPad Software, San Diego, CA).

### Individualized growth assessment

IGA was assessed using the publicly available iGAP software package (http://iGAP.research.bcm.edu), which allows the prediction of third-trimester growth trajectories and birthweight based on a Rossavik model of fetal growth. Growth models for HC, AC, femur diaphysis length, thigh circumference, and EFW (BPD–AC–TVol) are based on linear trends of the corresponding second-trimester growth curves. Each individual growth curve was based on a minimum of 2 second-trimester scans <28 weeks separated by 4-8 weeks. These velocity values were then compared to reference ranges previously calculated in a cohort of fetuses with normal growth outcomes.^28^ Participants (n = 0) delivering before their 34- to 36-week scan were excluded. Expected and observed measurements at 34-36 weeks were compared as follows: percent deviation = 100 × (observed-predicted)/predicted.

The mean of this percent deviation was taken as a measure of the systematic prediction error and its SD as a measure of the random prediction error^43^ among normally grown infants in this cohort. The modified Prenatal Growth Assessment Score (mPGAS), a composite growth index that combines the percent deviations of HC, AC, femur diaphysis length, thigh circumference, and EFW (BPD–AC–TVol), was calculated for each fetal growth curve. The negative version of mPGAS was used to classify fetuses with failing growth potential. Fetuses outside the previously reported 95% reference range of 0% to –0.17%^44^ were retrospectively identified as having abnormal growth. The growth potential realization index for birthweight (GPRIWT) is the ratio of the actual birthweight to the predicted birthweight multiplied by 100 and was calculated using measured birthweight within 24 hours of delivery and the predicted birthweight from the third-trimester growth curve (Supplementary Table 2). The predicted gestation at delivery is at the actual gestation if delivery is ≤38 weeks or at 38 weeks for deliveries occurring >38 weeks.^44^ The 95% reference range for this measurement is 84-118%; infants outside of this range were also defined as having abnormal growth following birth.^45^

The GROW customized birthweight centile and IGA were both included to enable an assessment of the agreement between the 2 methods. The GROW method uses final birthweight only and thus defines FGR by this parameter alone (adjusted for population effects of maternal characteristics and fetal sex),^46^ whereas IGA takes into account the individual growth potential of the fetus.

## Results

Clinical characteristics of the 115 participants are shown in Table 1. In the normal birthweight group with an IBR >10th centile (n = 91), there was an unexplained stillbirth and a preterm delivery at 36 weeks. Three babies were classified as large for gestational age with an IBR >90th centile. A total of 24 babies (21%) were SGA and 10 babies (9%) were FGR. The study was not powered to assess perinatal outcomes; however, there were no cesarean deliveries for nonreassuring fetal status in the normal birthweight group and 2 each in both the SGA and FGR groups. There were 3 admissions to neonatal intensive care unit in the normal birthweight group and 1 each in the FGR and SGA groups. There were no cases of neonatal metabolic acidosis.

**Table 1:** Baseline demographics of study participants (n = 115)

	IBR >10th centile, n = 91	FGR, n = 10	SGA, n = 24	*P* value
Maternal weight, kg	68.6 (59–74)	63.5 (61–74)	61.8 (58.8–70.5)	.48
Maternal height, cm	164 (160–169)	164 (158–168)	165 (161–168)	.91
Ethnicity				
European	73 (80)	10 (100)	24 (100)	.02
Indian	2 (2.2)			
Pakistani	4 (4.4)			
Caribbean	4 (4.4)			
Chinese	1 (1.1)			
Mixed other	2 (2.2)			
Mixed Asian-European	3 (3.3)			
Mixed Caribbean-European	2 (2.2)			
Nulliparous	59 (65)	5 (50)	12 (50)	.32
Birthweight, g	3525 (3200–3720)	2435 (2048–2720)	2733 (2455–2930)	<.001
Birthweight percentile	48.9 (29.1–74.1)	1.5 (1.0–2.2)	4.7 (2.0–8.2)	<.001
Gestational age at delivery, d	280 (273–287)	268 (262–277)	279 (267–289)	.02
Last scan to delivery interval, wk	5.3 (4.1–6.1)	2.8 (2.3–4.9)	4.9 (2.9–6.5)	.006

Data are presented as median (interquartile range) or number (%) unless otherwise specified.

*P* < .05 is significant; continuous variables were compared using Kruskal-Wallis test and categorical variables using Pearson χ^2^ test.

*FGR*, fetal growth restriction; *IBR*, individual birthweight ratio; *SGA*, small for gestational age.

*Simcox et al. Fractional thigh volume use in third-trimester fetal assessment. Am J Obstet Gynecol 2017*.

**Figure 2:**
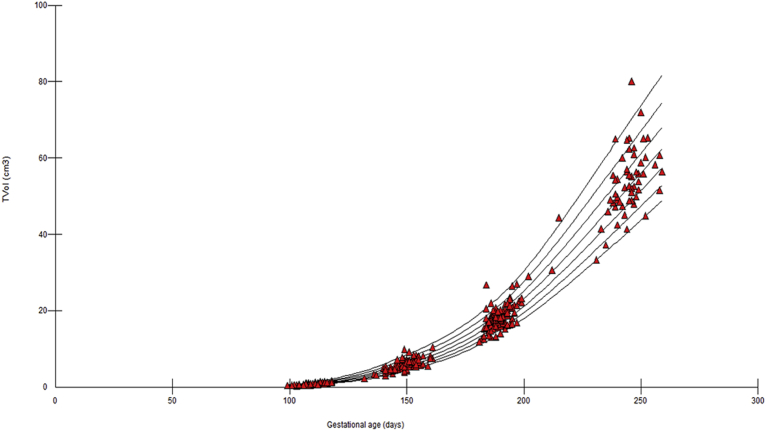
Scatterplots of fractional thigh volumes Scatterplots of fractional thigh volumes (TVol) (n = 357) during second and third trimesters of pregnancy with fitted percentiles (5th, 10th, 25th, 50th, 75th, 90th, and 95th). *Simcox et al. Fractional thigh volume use in third-trimester fetal assessment. Am J Obstet Gynecol 2017*.

TVol measures were taken in all participants and were normally distributed. Figure 2 shows a scatterplot of TVol during the second and third trimesters with fitted percentiles (5th, 10th, 25th, 50th, 75th, 90th, and 95th). The values of the TVol centiles are given in Table 2 for reference.

**Table 2:** Centiles for fractional thigh volume during second and third trimesters of pregnancy, cm^3^

Gestational age, wk	5th	10th	25th	50th	75th	90th	95th
14	0.224	0.256	0.295	0.348	0.416	0.494	0.592
15	0.331	0.376	0.431	0.505	0.598	0.703	0.834
16	0.562	0.633	0.719	0.835	0.979	1.14	1.33
17	0.958	1.07	1.21	1.39	1.61	1.86	2.15
18	1.45	1.60	1.80	2.06	2.36	2.69	3.09
19	2.09	2.31	2.58	2.91	3.32	3.76	4.27
20	2.95	3.25	3.60	4.04	4.57	5.13	5.78
21	3.98	4.36	4.80	5.37	6.03	6.73	7.53
22	5.06	5.53	6.06	6.74	7.53	8.35	9.29
23	6.26	6.81	7.44	8.24	9.16	10.1	11.2
24	7.70	8.35	9.09	10.0	11.1	12.2	13.5
25	9.41	10.2	11.0	12.1	13.4	14.7	16.1
26	11.4	12.3	13.4	14.6	16.1	17.6	19.3
27	13.8	14.9	16.1	17.6	19.3	21.0	22.9
28	16.5	17.8	19.2	20.9	22.9	25.0	27.2
29	19.5	21.0	22.7	24.7	27.1	29.5	32.1
30	22.8	24.5	26.5	28.9	31.7	34.5	37.6
31	26.3	28.3	30.5	33.4	36.5	39.8	43.4
32	29.9	32.2	34.8	38.0	41.6	45.3	49.5
33	33.7	36.3	39.2	42.8	46.9	51.0	55.7
34	37.6	40.5	43.7	47.7	52.2	56.8	61.9
35	41.6	44.7	48.2	52.6	57.6	62.6	68.2
36	45.6	49.0	52.8	57.5	62.9	68.3	74.3
37	49.6	53.2	57.3	62.4	68.1	74.0	80.4

*Simcox et al. Fractional thigh volume use in third-trimester fetal assessment. Am J Obstet Gynecol 2017*.

### Prediction of birthweight using 2D and 3D ultrasound parameters

In isolation, 2D and 3D ultrasound measures at 26-28 weeks’ gestation were poor predictors of birthweight (Table 3). For this reason, biometry measured at 26-28 weeks was not assessed further in prediction models. The 34- to 36-week measurements were more closely correlated to birthweight: the highest R^2^ value being 0.76 for EFW (BPD–AC–TVol) compared to EFW (Hadlock) (R^2^ = 0.62); comparison of Akaike and Bayesian information criterion confirmed lower values, indicating a better model fit with EFW (BPD–AC–TVol).

**Table 3:** Correlation of different ultrasound parameters at 26–28 and 34–36 weeks to birthweight

Measure	26–28 wk			34–36 wk			
	Coefficient (95% CI)	*P* value	R^2^	Coefficient (95% CI)	*P* value	R^2^	AIC
BPD, mm	55.7 (27.1–84.4)	<.001	0.32	46.4 (23.6–69.1)	<.001	0.35	
HC, mm	26.1 (15.6–36.6)	<.001	0.37	19.3 (11.0–27.6)	<.001	0.38	
AC, mm	29.5 (20.8–38.2)	<.001	0.45	24.0 (18.9–29.0)	<.001	0.59	1659.17
FL, mm	108.4 (65.5–151.3)	<.001	0.39	50.7 (24.8–76.6)	<.001	0.35	1711.66
TVol, cm^3^	90.3 (59.3–121.3)	<.001	0.42	39.3 (32.9–45.7)	<.001	0.68	1628.74
EFW (Hadlock), g	3.00 (2.24–3.76)	<.001	0.51	1.36 (1.10–1.62)	<.001	0.62	1648.57
EFW (BPD–AC–TVol), g	2.79 (2.01–3.57)	<.001	0.49	1.57 (1.36–1.77)	<.001	0.76	1597.03

All linear regression models adjusted for gestational age at time of scan and birth.

AIC used to compare models confirming better model fit with EFW (BPD–AC–TVol).

*AC*, abdominal circumference; *AIC*, Akaike information criterion; *BPD*, biparietal diameter; *CI*, confidence interval; *EFW*, estimated fetal weight; *FL*, femur length; *HC*, head circumference; *TVol*, fractional thigh volume.

*Simcox et al. Fractional thigh volume use in third-trimester fetal assessment. Am J Obstet Gynecol 2017*.

Table 4 demonstrates the screening performance of ultrasound at 34-36 weeks using the 2 EFW formulae, TVol, and AC for the prediction of SGA and FGR at birth. For the prediction of SGA, the AUC values for EFW (Hadlock), EFW (BPD–AC–TVol), TVol, TVol centile, and AC were similar at 0.87, 0.91, 0.86, 0.86, and 0.85, respectively. The only significant difference between prediction models was between TVol and EFW (BPD–AC–TVol) (*P* = .04), but EFW (BPD–AC–TVol) was not significantly better than EFW (Hadlock).

**Table 4:** Screening performance for small for gestational age and fetal growth restriction based on fetal biometry and fractional thigh volume at 34–36 weeks

	SGA (<10th centile), n = 24							FGR (<3rd centile), n = 10						
	AUC	Sensitivity, %	Specificity, %	Positive likelihood ratio	Negative likelihood ratio	PPV, %	NPV, %	AUC	Sensitivity, %	Specificity, %	Positive likelihood ratio	Negative likelihood ratio	PPV, %	NPV, %
EFW (Hadlock)	0.87	50	95	9.1	0.53	71	88	0.91	50	98	26.3	0.51	71	95
EFW (BPD–AC–TVol)	0.91	58	92	7.6	0.45	67	89	0.94	40	98	21.0	0.61	67	95
AC	0.85	38	95	6.8	0.66	64	85	0.85	20	100	–	0.80	100	93
TVol	0.86	50	92	6.5	0.54	63	88	0.92	40	98	21.0	0.61	67	95

*AC*, abdominal circumference; *AUC*, area under receiver operating characteristic curve; *BPD*, biparietal diameter; *EFW*, estimated fetal weight; *FGR*, fetal growth restriction; *NPV*, negative predictive value; *PPV*, positive predictive value; *SGA*, small for gestational age. *TVol*, fractional thigh volume.

*Simcox et al. Fractional thigh volume use in third-trimester fetal assessment. Am J Obstet Gynecol 2017*.

**Figure 3:**
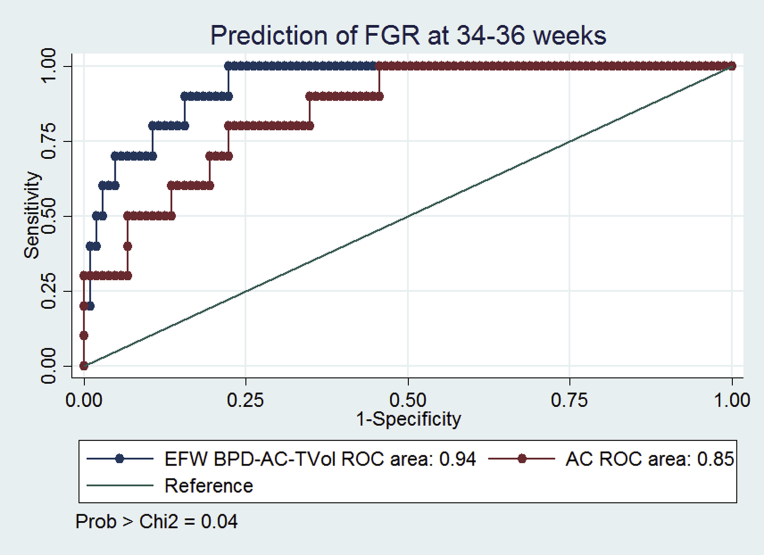
Area under ROC for ultrasound examination Area under receiver operating characteristic curve (ROC) for ultrasound examination at 34-36 weeks’ gestation for prediction of fetal growth restriction (FGR) using abdominal circumference (AC) and estimated fetal weight (EFW) (biparietal diameter [BPD]–AC–fractional thigh volume [TVol]) measurements. χ^2^ Statistic is used for comparison. *Simcox et al. Fractional thigh volume use in third-trimester fetal assessment. Am J Obstet Gynecol 2017*.

For the prediction of FGR, the AUC values were also similar apart from the AUC for EFW (BPD–AC–TVol), which was significantly greater than the AUC for AC alone (0.939 vs 0.847; *P* = .03) (Figure 3). For individual measurements, the specificity of TVol and AC were similar (98% vs 100%), but the sensitivity was higher for TVol (40% vs 20%). Comparison of TVol centile to the absolute TVol measurement did not improve the prediction.

### IGA protocol

IGA was evaluated using the iGAP tool. The mean percentage error for the Rossavik growth model of EFW (BPD–AC–TVol) was calculated for normally grown fetuses (excluding the above-described 15 FGR and 3 large-for-gestational-age fetuses, n = 97). There was a systematic error of –1.0% and a random prediction error of 6.1%. Three babies were identified as large by IGA with GPRIWT values >95th confidence interval and IBR values >90. Fifteen babies (13%) had abnormal mPGAS (n = 7), a GPRIWT for birthweight below the reference range (n = 2), or both (n = 6). Of those 15 infants, 12 had an IBR <10th centile (9 <3rd centile), but in 3 cases the IBRs were >15 (Table 5). Among the infants classified as FGR using the different definitions (IBR, mPGAS, and/or GPRIWT), 15/16 were abnormal using 2 criteria. Application of a <10th centile threshold for TVol or EFW (BPD–AC–TVol) at 34-36 weeks would have identified more infants with birthweights <3rd centile than with EFW (Hadlock) (7/10 vs 2/10; *P* = .07) (Table 5). There were 14 infants with a customized birthweight centile >3rd and <10th; of these, 3/14 had abnormal mPGAS and/or GPRIWT scores suggestive of true FGR. In addition, 1 baby IBR <3rd centile had normal mPGAS/GPRIWT/TVol, which may have indicated SGA rather than FGR. The prediction of FGR defined using mPGAS and/or GPRIWT is shown in Supplementary Table 1.

**Table 5:** Comparison of birthweight and ultrasound metrics for those infants defined as fetal growth restriction based on individual birthweight ratio <3rd centile, modified Prenatal Growth Assessment Score <–0.17%, and/or growth potential realization index for birthweight <84%

IBR	PGAS	GPRI	TVol centile	EFW centile	EFW TVol centile
0.0^a^	–1.4^a^	65.4^a^	0.4^a^	14.5	2.4^a^
0.9^a^	–1.0^a^	83.1^a^	0.3^a^	5.2^a^	1.0^a^
1.0^a^	–0.7^a^	80.9^a^	0.1^a^	12.6	1.2^a^
1.0^a^	0.0	92.9	24.0	7.8^a^	3.6^a^
1.2^a^	0.0	82.2^a^	12.0	27.6	5.6^a^
1.8^a^	–0.5^a^	81.3^a^	1.0^a^	35.5	13.7
2.2^a^	–1.7^a^	78.3^a^	1.0^a^	14.4	3.5^a^
2.2^a^	0.0	78.8^a^	31.0	30.8	16.5
2.3^a^	–2.7^a^	94.8	2.0^a^	19.0	3.5^a^
2.7^a^	–1.2^a^	85.6	6.0^a^	34.5	8.1^a^
5.5	–0.9^a^	94.4	3.0^a^	53.2	12.4
7.4	–0.9^a^	83.5^a^	2.0^a^	25.9	8.3^a^
8.9	–0.7^a^	100.5	0.2^a^	41.4	3.7^a^
18.1	–0.6^a^	98.6	1.0^a^	20.5	7.8^a^
18.8	–0.9^a^	116.8	2.0^a^	25.2	5.4^a^
44.7	–1.2^a^	99.9	13.0	70.2	50.0

*EFW*, estimated fetal weight; *GPRI*, growth potential realization index; *IBR*, individual birthweight ratio; *PGAS*, Prenatal Growth Assessment Score; *TVol*, fractional thigh volume.

*Simcox et al. Fractional thigh volume use in third-trimester fetal assessment. Am J Obstet Gynecol 2017*.

a Measurements below threshold demonstrating difference between different criteria. 34–36 week TVol threshold of <10th centile or EFW <10th centile (Gestation-Related Optimal Weight [GROW]) calculated using TVol would have identified 7/10 and 8/10 babies <3rd customized birthweight centile, respectively. In contrast to <10th centile (GROW) threshold using EFW (Hadlock) <10^th^, which would have identified 2/10.

## Comment

### Principal findings of the study

This study is the first to define reference TVol centiles across the third trimester of pregnancy using the LMS method. The fitted centiles from 18-37 weeks are similar to a previous study on TVol centiles using a different method.^32^ In our study at 20 weeks, measurements at the 5th, 50th and 95th centiles were 3.0 cm^3^, 4.0 cm^3^, and 5.8 cm^3^ and 45.6 cm^3^, 57.5 cm^3^ and 74.3 cm^3^ at 36 weeks, respectively. This compares with those previously reported (20 weeks: 3.7, 5.3, and 7.4 cm^3^; 36 weeks: 43.6, 62.6, and 89.9 cm^3^).^32^ We have also demonstrated that TVol is more closely correlated to birthweight than standard 2D measurements and comparable to 2D EFW and AC measurements for the detection of SGA and FGR at birth. In addition, this is also the first report of a clinical evaluation of the use of IGA in the prediction of SGA and FGR. TVol was better than AC for the prediction of FGR although the 2 EFW formulae were comparable for the prediction of FGR.

### Results in context

With regard to SGA and FGR prediction, few studies have examined the correlation between ultrasound and birthweight at 34-36 weeks with more focus on ultrasound EFW estimations within 7 days of delivery. Linear regression analysis of 2D and 3D ultrasound measures at 34-36 weeks demonstrated a good correlation with birthweight, especially for EFW (BPD–AC–TVol) (R^2^ = 0.76). This improves on the R^2^ values of 0.40 reported by Hindmarsh et al^36^ obtained with 2D biometry at 30 weeks. A previous study examined EFW (BPD–AC–TVol) at 34-36 weeks’ gestation in 125 gestational diabetic pregnancies and found it to be more accurate than EFW (Hadlock).^47^ Other studies predicting birthweight using 2D ultrasound EFW and fetal thigh volume reported similar R^2^ values for EFW (Hadlock) (R^2^ 0.42) at 37 weeks, but no significant association of TVol to birthweight.^48^, ^49^ However, the latter study was small (n = 42) and observer repeatability was not tested.^49^

Our results demonstrated a similar performance of TVol and 2D measurements at 34-36 weeks to that of Roma et al^41^ and Sovio et al.^50^ Roma et al^41^ obtained AUC at 36 weeks of 0.82 and 0.86 for the detection of FGR (birthweight <10th centile) and severe FGR (birthweight <3rd centile) using 2D EFW, with detection rates of 39% and 61%, respectively. Sovio et al^50^ reported AUC at 36 weeks of 0.87 and 0.91 for SGA <10th centile and severe SGA <3rd centile using 2D EFW, with sensitivities of 57% and 77%, respectively.

The use of iGAP software demonstrated low systematic and random prediction errors for EFW (BPD–AC–TVol) (–1.0% ± 6.1%), although the systematic error is slightly higher than previously published values using a similar method (0.12% vs 1.0%).^26^ However, in the study by Lee et al multiple regression analysis was required to determine model coefficients, whereas in the iGAP model coefficients are known but Rossavik models are needed to provide appropriate parameter values. In this study, we also used the individually derived parameters (mPGAS and/or GPRIWT outside 95% reference range for birthweight) to distinguish FGR from SGA, which has the potential advantage of including fetuses failing to grow appropriately born >10th centile. In this study, there was good agreement between the different classifications of fetal growth using IGA and GROW customized centiles; 9/10 infants born <3rd centile had abnormal scores, 11/14 SGA infants had abnormal scores, and 88/91 appropriate-for-gestational-age infants had normal scores. There were also 3 cases of macrosomia detected by IGA in agreement with macrosomia as defined by conventional customized birthweight centiles.

### Clinical implications

We previously demonstrated that TVol is highly reproducible in clinical practice^30^ and this holds true for measurements obtained late in the third trimester.^51^ Few studies evaluated the interobserver reliability of ultrasound-derived EFW^52^ but this can vary by as much as 15-20% from actual birthweight.^53^ Interobserver variability for HC and AC measurements also increases with gestation due to fetal position, reduced liquor, or fetal breathing movements that make obtaining correct ultrasound measurement planes difficult.^54^ With the advent of semiautomated measurement acquisition and good reproducibility, TVol may therefore be clinically useful in the detection of FGR in late pregnancy.^32^

Although small numbers limited our study, we have demonstrated that TVol alone is comparable to 2D biometry measures (EFW [Hadlock] and AC), for the detection of FGR fetuses at 34-36 weeks and demonstrated that sensitivity could perhaps be improved by the inclusion of this measurement. This may be related to exponential increase in soft-tissue mass >28-29 weeks in normally grown, but not FGR fetuses, which is reflected by TVol measurements (current data and Lee et al^32^).

### Research implications

Several studies demonstrated the potential value of IGA for the detection of pathological growth. A study by Deter et al,^55^ which examined the detection of FGR fetuses using a combination of birthweight, placental assessment, and IGA, found that 14/16 fetuses (87.5%) retrospectively classified as FGR had abnormal GPRIWT values for birthweight. More recently, another study by the same group concluded that on a retrospective analysis of 184 SGA fetuses the majority (67%) with both abnormal fetal pathology growth scores (similar to mPGAS) and negative GPRIWT values were <5th centile for birthweight.^56^ While the iGAP online software package is straightforward to use and could be introduced into clinical practice, further validation of the IGA method for the identification of late pregnancy FGR is required. Ideally this would include placental markers indicative of poor placental function FGR (eg, placental growth factor^57^, ^58^), histological examination of the placenta, and postnatal markers of FGR such as the presence or absence of neonatal catch-up growth.^59^, ^60^

Further work is also required to understand the importance of optimal fetal growth in the context of ongoing childhood development; this research can only progress with more accurate tools for the detection of abnormal fetal growth.^61^, ^62^

### Strengths and limitations

The strength of our study method is in the standardized image acquisition on a low-risk obstetric population by 2 investigators. This necessarily limited the size of the cohort studied such that differences in perinatal outcome could not be assessed, although previous studies have proven enhanced detection of FGR/SGA to be a desirable outcome.^3^, ^50^ In addition, although TVol measurements were concealed and analyzed without knowledge of the pregnancy outcome, the 2D biometry measurements results were made available to the sonographers and clinical care teams. This would have prompted further monitoring for cases of suspected SGA. Another limitation is that the distribution of TVol measurements used in generating the reference range were clustered around 100, 150, 175, and 250 days, rather than being uniformly distributed across gestation. Furthermore, in the current study, we were only able to evaluate 1 IGA parameter (GPRIWT) to classify fetuses with reduced growth potential. If neonatal measurements of thigh circumference had been available, it may have further improved our detection of FGR fetuses.^44^

### Conclusions

In conclusion, our study suggests that 3D TVol on its own is equivalent or better to 2D biometry for detecting SGA and FGR at 34-36 weeks’ gestation. Moreover, this study demonstrated the potential clinical utility of IGA for the identification of abnormal fetal growth. There are potential advantages in measurement acquisition, positive predictive value, and identification of at-risk fetuses that warrant larger scale study.

## Figures and Tables

**Supplementary Table 1:** Screening performance for fetal growth restriction <3rd centile and fetal growth restriction classified by individualized growth assessment methods at 34–36 weeks

	FGR (mPGAS and/or GPRIWT outside 95% reference range), n = 15						
	AUC	Sensitivity, %	Specificity, %	Positive likelihood ratio	Negative likelihood ratio	PPV, %	NPV, %
EFW (Hadlock)	0.90	27	96	6.7	0.76	50	90
EFW (BPD–AC–TVol)	0.94	53	97	17.8	0.48	73	93
AC	0.86	27	97	8.9	0.76	57	90
TVol	0.97	73	96	18.8	0.278	73	96

*AC*, abdominal circumference; *AUC*, area under receiver operating characteristic curve; *BPD*, biparietal diameter; *EFW*, estimated fetal weight; *FGR*, fetal growth restriction; *GPRIWT*, growth potential realization index for birthweight; *mPGAS*, modified Prenatal Growth Assessment Score; *NPV*, negative predictive value; *PPV*, positive predictive value; *TVol*, fractional thigh volume.

*Simcox et al. Fractional thigh volume use in third-trimester fetal assessment. Am J Obstet Gynecol 2017*.

**Supplementary Table 2:** Glossary of terms

Term	Definition
GPRI	IGA indicator of neonatal growth outcome for individual anatomical parameters GPRI = (measured birth parameter value/predicted parameter value) × 100
GPRIWT	GPRI for birthweight
mPGAS	Growth assessment score for third trimester that uses percent deviation values for HC, AC, femur diaphysis length, thigh circumference, and EFW (BPD–AC–TVol) to individual anatomical parameters at end of third trimester and combinations of anatomical parameters at individual time points Percent deviation = [(measured value-predicted value)/predicted value] × 100]
IGA	Method whereby each fetus acts as its own control Second-trimester growth velocity data are used to determine Rossavik growth models that predict individual third-trimester growth trajectories
iGAP	An online growth assessment program using IGA methods to calculate third-trimester predicted size trajectories and birth characteristics
IBR	Birthweights/fetal weights are assessed in reference to standard that is individually adjusted for physiological pregnancy variables (maternal height, weight, parity, and ethnic group) Postnatally, standard is also adjusted for sex of baby

*AC*, abdominal circumference; *BPD*, biparietal diameter; *EFW*, estimated fetal weight; *GPRI*, growth potential realization index; *HC*, head circumference; *IBR*, individual birthweight ratio; *IGA*, individualized growth assessment; *mPGAS*, modified Prenatal Growth Assessment Score; *TVol*, fractional thigh volume.

*Simcox et al. Fractional thigh volume use in third-trimester fetal assessment. Am J Obstet Gynecol 2017*.
